# The Mckittrick-Wheelock Syndrome: A Rare Cause of Severe Hydroelectrolyte Disorders and Acute Renal Failure

**DOI:** 10.1155/2011/765689

**Published:** 2011-10-01

**Authors:** Andrea Bruno, Domenico Chimienti, Alda Montanaro, Fernando Prete, Pasquale Libutti, Piero Lisi, Carlo Basile

**Affiliations:** ^1^Division of Nephrology, Miulli General Hospital, 70021 Acquaviva delle Fonti, Italy; ^2^Division of Surgery, Miulli General Hospital, 70021 Acquaviva delle Fonti, Italy

## Abstract

The McKittrick-Wheelock syndrome is a rare cause of severe hydroelectrolyte disorders and fluid depletion as a result of rectal tumor hypersecretion, which can lead to acute renal failure. We report the case of a 70-year-old female who presented with hyponatremia, hypokalemia, hypochloremia, and acute renal failure, due to a watery, mucinous diarrhea. A large rectal villous adenoma was discovered on ileocolonoscopy, and definitive management was achieved by removal of the tumor. In conclusion, reversal of the biochemical derangement is the cornerstone of successful management of the McKittrick-Wheelock syndrome. Then, immediate surgical resection of the tumor is the treatment of choice.

## 1. Introduction

Adenomatous polyps are neoplasms with malignant potential, located mainly at the level of the sigmoid and rectum. Most patients with colonic adenomatous polyps present with mild gastrointestinal symptoms or are asymptomatic, colonoscopic exploration being the procedure of choice for the diagnosis [1]. In rare cases, patients with villous adenomas exhibit secretory diarrhea with considerable loss of fluids and electrolytes. Secretory diarrhea is generally defined as any diarrheal disease in which intestinal fluid losses exceed 10 mL/kg body weight/day [2]. It is due to alterations in the transport of fluids and electrolytes through the intestinal mucosa. It is characterized clinically by abundant watery stools, typically not accompanied by pain, which persist despite fasting. Causes are either infectious (such as cholera) or noninfectious [2]. The rare McKittrick-Wheelock syndrome, first described in 1954, is characterized by severe fluid and electrolyte depletion secondary to mucous diarrhea from rectal tumors, most notably villous adenoma [3–8]. Cases of mucous hypersecretion from villous adenomas causing dehydration, hyponatremia, hypokalemia, and hypochloremia have been reported [3–8]. We present a case of McKittrick-Wheelock syndrome with severe biochemical derangements.

## 2. Case Report

A 70-year-old female presented to our emergency department. Her medical history documented arterial hypertension for which she was receiving a hypotensive drug treatment (no diuretics). The recent medical history showed that she had been hospitalized the previous month in a cardiology unit of another hospital and then transferred to the nephrology unit of the same hospital because of “complex ventricular extrasystoles due to hypokalemia caused by mushroom poisoning.” Two weeks after the discharge, she presented to our emergency department with symptoms of progressive weakness, drowsiness, and oliguria. She had the complaint of abundant loose stools for just a few days (above all at night). On examination, there was clinical evidence of volume depletion, with dry mucous membranes; her temperature was 36.6°C, her pulse 89 beats per minute, and her respiratory rate 18 breaths per minute, and her blood pressure 100/50 mmHg. Chest radiograph and renal ultrasound scan were normal. Blood testing revealed renal failure with serum urea 65 mg/dL and serum creatinine 1.8 mg/dL (Table 1). Serum and urinary biochemistry are shown in Table 1. Serum electrolytes were deranged with sodium 113 mmol/L, potassium 2.9 mmol/L, and chloride 72 mmol/L (Table 1). These were the reasons why she was admitted to our nephrology unit. The patient was volume-resuscitated with 10 L 0.9% saline plus potassium in the first three days. At day 5 after admission, serum biochemistry was normal (Table 1). On that day, 24-hour urinary electrolytes were sodium 8 mmoL, potassium 12 mmoL, and chloride 10 mmoL. A watery, mucinous diarrhea with bowel actions as frequent as 10 times a day restarted in the following days with new severe biochemical derangements at day 10 and normalization of the parameters at day 15 (Table 1). An ileocolonoscopy revealed a large ulcerated rectal villous adenoma, occupying 80% of the luminal circumference extending from the anal verge (but sparing the sphincter) to 11 cm into the rectum. Definitive management was achieved with an intersphincteric rectal resection via laparoscopic transanal pull through and side-to-end colo-anal anastomosis with covered ileostomy [6]. The operation was uneventful and the histologic examination showed an early rectal cancer (pT1pN1) with 1.5 of distal clearance. 

The followup at 1, 3, and 6 months showed a stable clinical situation with normal serum biochemistry: urea 12 mg/dL, creatinine 0.7 mg/dL, sodium 143 mmol/L, potassium 4.1 mmol/L, and chloride 106 mmol/L.

## 3. Discussion

Villous colorectal adenomas are common tumors that normally provoke scarce symptomatology. The McKittrick-Wheelock syndrome is a rare cause of secretory diarrhea caused by a colorectal adenoma, a common tumor of this part of the intestine, which normally shows poor symptoms, thus frequently leading gastroenterologists and/or nephrologists to inappropriate diagnoses. Characteristically, there is watery, mucinous diarrhea with bowel actions as frequent as 20 times a day, not uncommonly up to 15 years prior to recognition of the cause. At the outset, the fluid and electrolyte losses are easily compensated for by increased oral intake and renal regulation. As the tumor size increases, these losses overwhelm compensatory mechanisms and the patient may seek medical attention [9]. Resulting losses can amount to 1.5–3.5 L of fluid containing 40–160 mmol/L sodium, 15–105 mmol/L potassium, and 80–165 mmol/L chloride [10]. The mechanism of fluid and electrolyte loss is unclear. Local release of prostaglandin E_2_ has been suggested as the secretagogue responsible for salt wasting; consequently, indomethacin and other prostaglandin inhibitors have been used with apparent benefit in controlling the volume of rectal effluent in patients with secretory villous adenomas [11, 12]. Unless complications arise due to severe hydro-electrolyte disorders, the McKittrick-Wheelock syndrome is a reversible illness with adequate treatment; thus, it is very important to make the diagnosis in precocious phases [4]. Despite all the warnings, the McKittrick-Wheelock syndrome can result in renal failure that requires hemodialysis, usually because of a delay in the diagnosis [5].

In conclusion, the McKittrick-Wheelock syndrome is a rare cause of severe hydroelectrolyte disorders and fluid depletion as a result of rectal tumor hypersecretion, which can lead to acute renal failure. Reversal of the biochemical derangement is the cornerstone of successful management. Then, immediate surgical resection of the tumor is the treatment of choice.

## Figures and Tables

**Table 1:** Serum and urinary biochemistry on admission and during patient's hospitalization.

	Admission	2 days	5 days	10 days	15 days
Serum sodium (mmol/L)	113	126	138	119	136
Urinary sodium (mmol/day)	15	89	102	21	123
Serum potassium (mmol/L)	2.9	3.4	3.8	2.6	3.8
Urinary potassium (mmol/day)	7	22	43	8	49
Serum chloride (mmol/L)	72	92	101	81	105
Serum calcium (mg/dL)	9.9	8.7	8.8	9.5	9.0
Serum urea (mg/dL)	65	46	17	59	14
Serum creatinine (mg/dL)	1.8	1.1	0.8	2.7	0.7
Serum glucose (mg/dL)	125	112	93	114	80

## References

[1] Bond JH (2000). Polyp guideline: diagnosis, treatment, and surveillance for patients with colorectal polyps. *American Journal of Gastroenterology*.

[2] Carpenter CCJ (1982). The pathophysiology of secretory diarrheas. *Medical Clinics of North America*.

[3] Lepur D, Klinar I, Miše B, Himbele J, Vranjican Z, Baršić B (2006). McKittrick-Wheelock syndrome: a rare cause of diarrhoea. *European Journal of Gastroenterology and Hepatology*.

[4] Martins HS, Brandão-Neto RA, de Carvalho AL (2007). McKittrick-Wheelock syndrome: a cause of severe hydro-electrolyte disorders in ED. *American Journal of Emergency Medicine*.

[5] Popescu A, Orban-Şchiopu AM, Becheanu G, Diculescu M (2005). McKittrick-Wheelock syndrome—a rare cause of acute renal failure. *Romanian Journal of Gastroenterology*.

[6] Targarona EM, Hernandez PM, Balague C (2008). McKittrick-Wheelock syndrome treated by laparoscopy: report of 3 cases. *Surgical Laparoscopy, Endoscopy and Percutaneous Techniques*.

[7] Winstanley V, Little MA, Wadsworth C, Cohen P, Martin NM (2008). The McKittrick-Wheelock syndrome: a case of acute renal failure due to neoplastic cholera. *Renal Failure*.

[8] Miles LF, Wakeman CJ, Farmer KC (2010). Giant villous adenoma presenting as McKittrick-Wheelock syndrome and pseudo-obstruction. *Medical Journal of Australia*.

[9] Ashman N, Yaqoob M (2000). Metabolic acidosis, hypokalaemia and acute renal failure with a normal urine output. *Nephrology Dialysis Transplantation*.

[10] DaCruz GMG, Gardner JD, Peskin GW (1968). Maechanism of diarrhea of villous adenomas. *The American Journal of Surgery*.

[11] Pugh S, Thomas GAO (1994). Patients with adenomatous polyps and carcinomas have increased colonic mucosal prostaglandin E2. *Gut*.

[12] Smelt AHM, Meinders AE, Hoekman K, Noort WA, Keirse MJNC (1992). Secretory diarrhea in villous adenoma of rectum: effect of treatment with somatostatin and indomethacin. *Prostaglandins*.

